# A century of suicide: Insights from long-term data in the United States

**DOI:** 10.1073/pnas.2519951123

**Published:** 2026-04-27

**Authors:** Nina de Lacy, Wai-yin Lam, Timothy Collins, David Danks, Fernando A. Wilson, Ken R. Smith, Bernice A. Pescosolido

**Affiliations:** ^a^Department of Psychiatry, University of Utah, Salt Lake City, UT 84112; ^b^Huntsman Mental Health Institute, University of Utah Health Hospitals & Clinics, Salt Lake City, UT 84108; ^c^School of Environment, Society, and Sustainability, University of Utah, Salt Lake City, UT 84112; ^d^Corcoran Department of Philosophy, and School of Data Science, University of Virginia, Charlottesville, VA 22904; ^e^Matheson Center for Health Care Studies, University of Utah, Salt Lake City, UT 84108; ^f^Department of Family and Consumer Studies, University of Utah, Salt Lake City, UT 84112; ^g^Huntsman Cancer Institute, University of Utah Health Hospitals & Clinics, Salt Lake City, UT 84112; ^h^Department of Sociology, Indiana University, Bloomington, IN 47405; ^i^Irsay Institute, IU Research, Indiana University, Bloomington, IN 47405

**Keywords:** suicide, sociodemographic risk factors, historical epidemiology

## Abstract

The 2024 National Strategy for Suicide Prevention identifies suicide as “an urgent and growing public health crisis” driven partly by mental health and overdose rates, the COVID-19 pandemic, and declining social connectedness. Yet, data constraints have limited long-term investigations of suicide trends. Acknowledging limits in data sequence continuity, we constructed the Suicide Trends and Archival Comparative Knowledgebase (STACK), harmonizing data from 1900–2021. Coupling visualizations with Joinpoint regressions, we examine the contemporary suicide crisis in historical perspective. Data reveal patterns that both support and challenge prevailing understandings of suicide risk, trends, and drivers. Overall, we document a cyclical, dampening, and downward-trending pattern of suicide over 120 y, contrasting sharply with trends in other causes of death. However, this trend is offset by a sharp rise and unique patterning for persons under 35, beginning in the mid-to-late 1950s. The contemporary “youth crisis” spans a broader age range and begins much earlier than typically acknowledged. Each successive generation faces risk at younger ages, accompanied by a startling rise in suicide by hanging since the 1980s among both males and females. Despite ongoing concerns about rural populations and the elderly, living in large metropolitan areas (versus suburban and rural areas) and being older have recently emerged as protective factors. While analyses by race or rurality remain limited to the last half century because of data availability, the long-term temporal, geographic, and sociodemographic complexity of suicide mortality trends offers critical insights and intriguing avenues for next-generation research, treatment, and prevention efforts.

## Significance

High and rising suicide rates are a defining feature of the current US mental health crisis. Yet, the long-term historical patterning of US suicide rates remains poorly understood, especially across sociodemographic groups. This limits insights into mechanistic drivers of suicide inflection points and cohort dynamics, hindering evaluations of interventions, policy shifts, and social transitions. STACK data (1900-present) address this gap. Using visualization and Joinpoint regression, we offer insights that challenge established findings and reinforce emerging cohort-based models of suicide risk. These findings can help guide research priorities, refine causal and counterfactual modeling, and underscore the importance of relative intervention impacts. Significantly, they demonstrate how identifying inflection points can improve forecasting and optimize the timing of prevention efforts.

Making the argument for suicide as a collective phenomenon, and not simply the sum of personal troubles or genetic inheritance, sociologist Emile Durkheim maintained that societies “give up” a relatively stable quota of suicides that reflects *sui generis* forces (1). Temporal changes in suicide rates indicate that society itself is changing in the way it impacts individual lives and deaths. In the contemporary United States, one of the most pressing concerns lies in suicide’s contribution, along with drug overdoses, chronic diseases, and COVID-19, to the initial stagnation and now recent decline in life expectancy (2). With estimates of a nearly 40% rate increase since 2000, suicide ranks near the top of cause-specific mortality rates for middle-aged adults (4th for those aged 35–54) and, more surprisingly, for youth (2nd for those aged 10–34) (3, 4).

Until recently, factors such as sex (males), age (the oldest), marital status (unmarried/divorced), and socioeconomic factors (educational attainment, unemployed status) have been documented as fairly robust correlates with suicide risk at both the individual and geographic levels (5). These at-risk groups have been seen as consistent targets for prevention and treatment efforts by policymakers. Others have responded with consternation that our ability to predict suicide and quantify individual suicide risk has not substantively improved in the last 50 y (6). While some have proposed a reevaluation of candidate risk factors, (4) recent research has offered other potential directions. Confounding by period or cohort effects may be partially responsible for inconsistent results with respect to central issues such as unemployment (7, 8). In fact, a growing body of research supports the role of contextual heterogeneity in suicide deaths, with more recent research demonstrating that such effects persist even in the presence of individual-level risk factors (9).

Suicide’s remarkable resistance to certain individually focused preventive interventions, the periodicity of suicide risk, and marked changes in which groups are most at-risk, all support calls for research on the shifting landscape of suicide to inform novel prevention and treatment approaches (10). Despite this research need, prior analysis of temporal change in suicide has been limited to narrow historical periods, with incomplete characterization of how longer-term historic patterns compare to contemporary risks, especially across sociodemographic groups. Such analyses have been constrained by limited high-quality population suicide data with detailed covariates, coupled with revisions of death classification, and a changing complex patchwork of US medicolegal offices. However, existing data sources can be leveraged to identify changing patterns of US suicides, especially with detailed information for the last 50 y (11). The resulting analyses may 1) provide a deeper understanding of how historical context drives suicide risks, 2) identify which sociodemographic effects persist over time, and 3) reveal new emergent risks. The STACK is a dataset that integrates 122 y of US mortality data, drawing from National Vital Statistics Reports (NCHS, 1900–1968) and restricted, individual-level death certificate data (1968–2021). It provides consistent measure of cause of death, allowing long-term, descriptive analyses of trend across basic demographic subgroups (i.e., age, sex, race, cohort, place) to deepen our understanding of suicide risk and its context.

## Methods and Materials

Data on cause-specific mortality are drawn from two sources (12, 13). First, we extracted summary vital statistics data from annual reports (1900–1968) available on the National Center for Health Statistics (NCHS) website (14). Second, we used approved, restricted, deidentified, individual-level death certificate data obtained from Detailed Multiple Cause of Death (MCOD) files provided by NCHS for deaths from 1968–2021 (15). These MCOD data were made available through an agreement with NCHS that allowed use of microdata that were geocoded at the county level. Together, these two sources provided 122 y of the necessary cause-of-death data. In STACK, each record has variables describing numerous demographic characteristics of the deceased individual as well as the underlying cause of death. To generate suicide rates with MCOD data, we used population denominator counts for calendar year-sex-race-county-specific single-year age groups obtained from the Surveillance, Epidemiology, and End Results (SEER) Program that curates and distributes these US Census data (16). This study was deemed Not Human Subjects research by the University of Utah Institutional Review Board.

Suicides were identified in the NCHS files by International Statistical Classification of Diseases (ICD) codes. Multiple revisions of ICD codes were used in the MCOD data between 1900 and 2021. For 1900 through 1967 encompassing ICD-1 to ICD-7, deaths from suicide were identified by the code “Group XIII (Violence, Suicide)” for ICD-1, codes 155-163 for ICD-2, codes 165–174 for ICD-3, codes 163–171 for ICD-4, codes 163–164 for ICD-5, codes E970-E979 for ICD-6, and codes E963/ E970-E979 for ICD-7. For subsequent years where the microlevel MCOD are available, suicides were identified by codes E950-E959 for ICD-8 (1969–1978) and similarly for ICD-9 (1979–1998). In 1999–2021, only death records coded as U03, X60-X84, or Y87 using ICD-10 were identified as suicides. Only data from residents in the 50 states or District of Columbia were available (yearly sample sizes available, *SI Appendix*, Table S1). The total sample size of suicide deaths obtained from MCOD for 1969–2021 was 1,739,992 and the total sample size obtained from NCHS for 1900–1968 was 985,501. Of course, suicide rates have long been the subject of criticism, and issues of reliability and validity are only compounded when considered over time due to changes in ICD codes, professional standards, and cultural meanings (17). However, both specific studies as well as systematic analyses of rates, changes in ICD codes, and differences in qualifications of medicolegal officials conclude that suicide rates are likely underreported but do not appear to interfere with the analyses of the correlates of suicide rates (18–23).

The *crude suicide rate* of a given group i (e.g., males) is $\frac{n_{i}}{p_{i}}$, where $n_{i}$ and $p_{i}$ are the suicide deaths and population size (in 100,000) for group i, respectively. Age-standardized suicide rates were computed using direct standardization. Consider any age group j in 11 age categories (i.e., under 1, 1–4, 5–14, …, 75–84, 85 and over). Let $r_{j}$ be the age-specific suicide rate of age group *j* in the population of interest, $p_{j}$ be the population size in the *standard* population of age group j (e.g., US population in the year 2000), and P be the total population size of all age groups in the standard population. The age-standardized suicide rate is a weighted sum defined by $\sum_{j=1}^{11}\left(r_{j}\times \frac{p_{j}}{P}\right)$ (see exact values of the fraction $\frac{p_{j}}{P}$ from Hoyert & Anderson) (24).

Key sociodemographic subgroups used in understanding suicide, and available in STACK, are our analytic focus. They are defined by age, sex, and race from the mortality data and SEER population counts from the US Census. Additional characteristics used include birth cohort (i.e., generation), rurality of residence, and method of suicide death. Given unavoidable limitations at the point of data collection, race and method of death statistics are only available from 1969 and degree of rurality from 1974. Despite this limitation, we include both factors to fully develop insights from STACK data. For birth cohort, we adopt Dimock’s cohort classification (25). Records were identified by their generational cohort based on birth years: Silent Generation (1928–1945), Baby Boomers (1946–1964), Generation X (1965–1980), Millennials (1981–1996), and Generation Z (1997–2012). For rurality, Rural-Urban Continuum Codes (RUCC) were used to classify the residential county of each record. Three classes are considered in this work: Metro counties (RUCC = 0, 1, 2, 3), urban counties (RUCC = 4, 5, 6, 7), and rural counties (RUCC = 8, 9), using the definitions for the given year of analysis. While the publicly available SEER data span the years 1969–2022, racial classifications (i.e., White, Black, American Indian, Asian, or Pacific Islander) are available only from 2003–2020. Finally, suicide methods were classified into a) firearms or explosives, b) poisoning, c) hanging, strangulation, or suffocation (collectively, “hanging”), and d) others (associated ICD codes, *SI Appendix*, Table S2).

Analyses were done in stages and guided by the availability of consistent data for consecutive years of coverage. Analyses are longitudinal, presenting temporal patterns of suicide rates using two approaches. First, crude and age-standardized rates are shown over time. Second, indexed rates are shown based on a common starting point of time. Indexed rates allow suicide rates to be more easily compared in terms of magnitude of change over the course of time relative to a baseline year. Given a time period $t_{0},\cdots ,t_{n}$ and its corresponding set of crude/age-standardized rates $r_{0},\cdots ,r_{n}$, the indexed rate for each time point 0≤k≤n is defined by $r_{k}\times \frac{100}{r_{0}}$ where $t_{0}$ is the baseline standard time point.

We deployed Joinpoint regressions to identify significant shifts in time trends by estimating a series of linear segments (similar to splines) connected by “joinpoints” (or knots) (26). Selecting best joinpoint models was done by permutation testing and application of the Weighted Bayesian Information Criterion (WBIC), implemented by National Cancer Institute software (27, 28) The maximum number of potential joinpoints were specified and the best-fitting model was chosen without adjusting for the potential effects of covariates.

Finally, we estimated the number of preventable suicides under a best-case scenario. A simple simulation identified the lowest suicide rate for each of the years 1969–2021 for each age group. A complete set of the lowest age-specific suicide rates from 1969–2021 were applied to the age distribution for each year. This created annual synthetic suicide counts under the assumption that the lowest age-specific suicide rates ever observed applied in that year. Across years, we compared the number of observed suicides to the number of synthetic suicides. This generated an estimate of the number of suicides that would have been prevented if the lowest age-specific suicide rates were in place for all years.

## Results

### Suicide Mortality Displays an Unusual Cyclical Pattern over the Long Term.

**Fig. 1.**
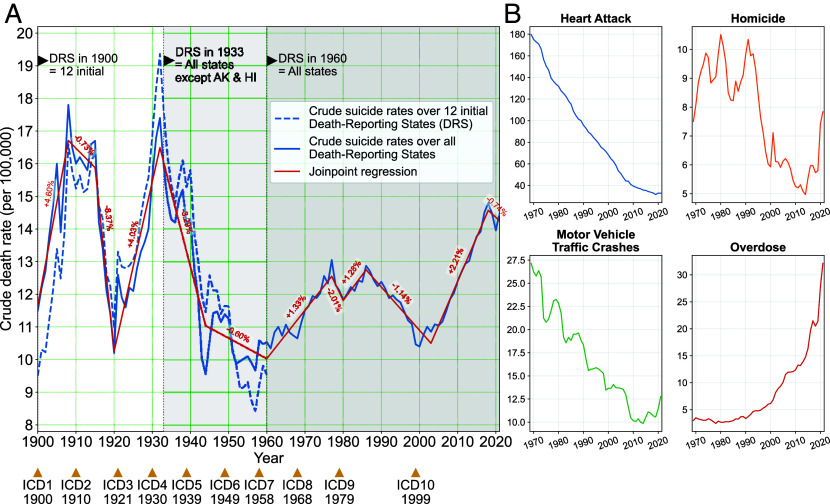
Annotated US crude suicide rates, 1900–2021. Multiple states joined or left the reporting system in 1900–1933. (*A*) shows crude suicide rates for all states reporting deaths at time of reporting (—) as well as the 12 Death-Reporting States (DRS) that were constant through 1933 (- - -). Years when successive International Classification of Disease (ICD) schema were adopted in the United States are shown at the bottom of a with results from joinpoint regression analysis in red. Figures in red on joinpoint trend lines are the percentage annualized change (deaths per 100,000 per year) in suicide in the relevant segment. (*B*) displays trends from 1970–2021 in other causes of death in the STACK. ICD codes used in encoding different causes of death can be found in *SI Appendix*, Table S2. The values of absolute annualized change and percentage change of the segments of the joinpoint regression trendline can be found in *SI Appendix*, Table S3.

Fig. 1 presents crude suicide rates over time. Crude rates are more sensitive to temporal fluctuation (age-standardized rates provided in *SI Appendix*, Fig. S1).^*^ Because early data only include geographic areas that were US states at that time, we provide a comparison of US suicide rates from 1900 onward (solid blue line) with those in the 12 founding states (dotted blue line). Results indicate that the variability in suicide rates is not attributable to the changing composition of the states that reported cause of death statistics.

While media reports often note that US suicide is at an all-time high, (29) this holds only for the absolute number of deaths, an artifact of population growth (*SI Appendix*, Fig. S1) or for rate comparison made across the more complete CDC suicide data since 1960 that is often sampled from the CDC WONDER resource. Fig. 1*A* instead reveals several striking patterns since 1900. First, suicide rates are currently quite high, but not at their highest. The maxima occurred during the Progressive Era (1910s) and Great Depression (1930s). Second, a minimum suicide rate of 9 to 11 deaths per 100,000 occurs multiple times across the century suggesting a potential lower bound on US suicide rates. Third, joinpoint regression analysis (red) shows that major upward trends in US suicide deaths began in 1920, 1960, and 2003 with a minor upward trend in 1980. Similarly, significant downward trends began in 1908, 1932, and 1986 with a minor downward trend in 1977. A new downward trend appears in 2017, although it is unclear whether this will persist, especially in the aftermath of the COVID-19 pandemic (4).

Suicide appears to be a cyclical phenomenon that reverses every 10 to 25 y with a dampening oscillation over time. This is a relatively unusual pattern in mortality data (Fig. 1*B*) based on the MCOD data. For instance, mortality rates from colorectal cancer and myocardial infarctions reveal consistently declining curves with moderate slopes from 1970 to 2020 reflecting progress made over the 20th century. This pattern is typical among common chronic diseases that have benefitted from improved screening and treatment. Motor vehicle accident deaths show step declines that reflect the impact of discrete, successful public health interventions (e.g., seat belt laws) and manufacturing safety improvements. Mortality rates from drug overdose exemplify the outbreak phase of an epidemic, similar to other crises such as HIV/AIDS or COVID-19. In contrast, cause-specific mortality rates with cyclical patterns, like the suicide rate, are rare. Incidence rates of infectious disease (e.g., tuberculosis, sexually transmitted diseases) may reveal similar trends because they are influenced by shifts in stigma, drug resistance, testing availability, or health policy. To our knowledge, the only other common mortality outcome that shows a similar cyclical pattern is homicide, another poorly understood cause of death with a complex etiology.

Given the large variability in rates over time, we estimated the total number of preventable suicides by comparing the observed number of suicides between 1969–2021 to the number expected if the lowest age-specific rates occurred for all years. The total observed number is 1,739,128 suicides, and the number of simulated suicides is 1,366,763 deaths. This suggests that 372,365 suicides (21.4% reduction) could have been prevented between 1969–2021 if each year had the lowest observed annual age-specific suicide rates.

### Risk for Younger Age Groups Markedly Accelerated Beginning in the Mid-1950s.

**Fig. 2.**
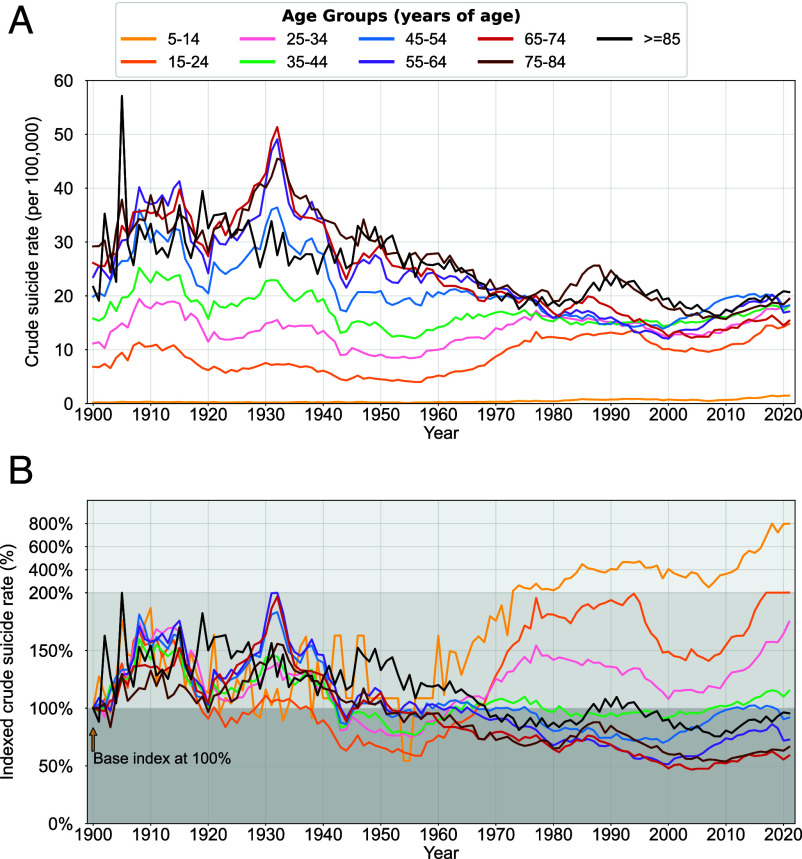
Crude suicide rates and indexed crude suicide rates for different age groups, 1900–2021 Crude suicide rates are shown in *A* for nine age groups from 1900 to 2021. In *B*, the same rates are indexed, transforming values so that the reference year’s value (1900) is set to a baseline, allowing for comparison of relative changes over time.

Historically, suicide risk was considered to be highest among older individuals. Fig. 2*A* shows that crude suicide rates between the oldest and youngest population groups differed by an order of magnitude during the first half of the 20th century. These gaps began to narrow later in the century, driven by simultaneously decreasing suicide rates among older ages and increasing rates for younger ages. By the late 1970s, a clear convergence across all age groups is evident. Indexing rates (Fig. 2*B*) allows a comparison of changes in relative trends among different age groups over time by setting a common starting point. Mortality rates indexed to 1900 appear to approximate a supermartingale for all groups over 35 y old—specifically, a general downward trend with declines in year-on-year volatility. However, suicide rates for the three youngest age groups (5–14, 15–24, 25–34 y) accelerated sharply, becoming much more variable after a marked inflection point (mid-1950s) and concomitant with the end of the long downward trend cycle (Fig. 1*A*) starting with the New Deal and ending in 1960. Despite a relative slowing in the 2000s, the trend of increasing rates reappears among younger age groups and accelerates to the present.

### Marked Cohort Effects Are Visible in the Death Risk by Suicide.

**Fig. 3.**
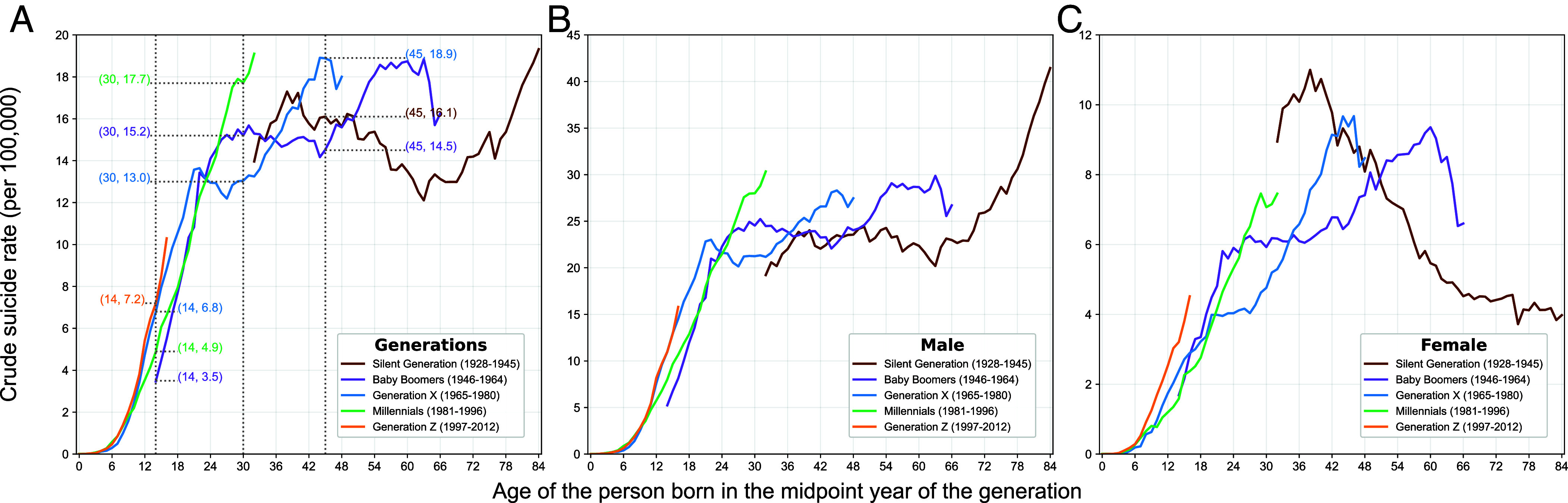
Crude suicide rates for five generational cohorts. Each trendline in this chart represents the crude suicide rate trajectory for individuals born in the midpoint year of each generation plotted against their age from 0 to 84 y. To facilitate comparison of suicide rates among cohorts, the numbers in parentheses adjacent to the lines indicate the crude suicide rate at ages 14, 30, and 45 y for each cohort. For example, a value of 17.7 at age 30 for Generation X means that 17.7 individuals per 100,000 in that cohort died by suicide at age 30. Panel (*A*) shows rates for both sexes combined, Panel (*B*) for males, and Panel (*C*) for females.

Studying differential suicide risk patterns associated with birth cohorts helps to identify potential effects of unique environmental, social, and economic conditions tied to specific historical periods (30–32). Fig. 3 shows sex-cohort-specific suicide risks since 1900 among five major birth cohorts whose suicide data were observed in the MCOD data, expanding on and supporting the analyses by Phillips (8). To help interpret these patterns, we first focus on an example comparison: How 30-year-old males differ across cohorts in their suicide risk (vertical dotted line at age 30). A 30-year-old Millennial male has a higher suicide risk than a 30-year-old Baby Boomer, and both are substantially higher than a 30-year-old Generation X-er. Indeed, at age 30, Millennials have a suicide rate that exceed by a third that of Gen-Xers. While the increases in suicide rates at younger ages have similar patterns across successive cohorts, they diverge at age 25. Cohort differences are also highlighted at other younger (age 14) and older (age 45) ages. For females, there are notable cohort differences in relation to males. For those under age 18, Gen-Z females have experienced a steeper increase up to age 18 in suicide risk compared to Millennials and Gen-Xers. For earlier birth cohorts, we find that female suicide rates reach their zenith during middle age, though earlier for the Silent Generation (age 38) and later for Generation X (age 46) and the Silent Generation (age 62). Declines in suicide rates after these peaks are distinctive and not observed among their male cohort counterparts. Comparing female and male members of the Silent Generation reveals a vivid bifurcation where male rates steadily rise starting at age 62 while for females, they started to decline at age 38, which continued largely unabated to the present.

These cohort patterns highlight the influence of unique historical factors (i.e., period effects) experienced by each cohort and how they differentially shape suicide risks by sex. Moreover, these results underscore the fact that age patterns in suicide risk are not static but are sensitive to the varying social forces that serve to uniquely define the distinct lived experiences of each cohort. For the Baby Boomer generation, for males and females alike, the trajectory of suicide rates steadily increase to about age 20 but then plateaus for the next 20 y at which point the increases resume, but then decline again in their 60 s. Baby Boomers faced many challenges while in their 20 s and 30 s due to the competitiveness brought on by their large cohort size, (33) the Vietnam War, shifts in the political zeitgeist due to the Watergate Era, and the recessions centered around 1975 and 1982. While tumultuous, these period effects were surprisingly associated with a decelerating suicide rate. In contrast, the Millennial Generation did not experience a similar inflection point starting in their 20 s but instead have suicide rates that continue to rise up to the present. They too faced challenges engendered by period effects but attributable to rising political discord and polarization—historical conditions that differed in their effect relative to those of the Baby Boomers. And finally, for the most dramatic shifts in suicide, the age patterns for the Silent Generation showed marked and distinctive patterns by sex. As noted, the rise in rates for men began around age 62 that coincide with the years 1990–2007 and increase almost unabated from 2012 to the present. In contrast, the steady decline in suicide for women starts younger at age 38 coinciding with the years 1965–1982. While many forces are potentially at play, the effects on men may reflect the cumulative effects of several wars and poor health exacerbated by elevated tobacco use. For women of this generation, they were the mothers that gave rise to the Baby Boom generation and the lifelong impact of raising children and the possible support they provide to these women may have contributed to their declining rates of suicide.

### Suicide Rates for Females Departed from Long-Standing Patterns in the Mid-1950s.

**Fig. 4.**
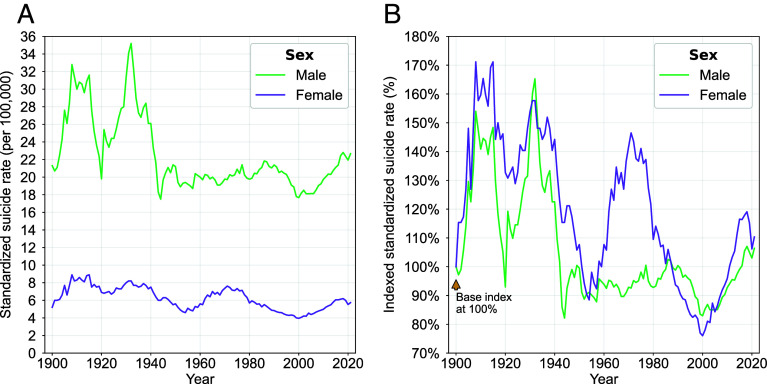
Age-standardized and indexed suicide rates by sex, 1900–2021 Age-standardized suicide rates are shown in *A* for males and females from 1900 to 2021. In *B*, the same rates are indexed, transforming values so that the reference year’s value (1900) is set to a baseline, allowing for comparison of relative changes over time.

One of the most durable observations in US suicide research finds age-standardized suicide rates considerably higher for males than females (Fig. 4*A*) (34). Male long-term rates resemble overall population trends (Fig. 1), particularly in the first half of the 20th century. Indexed rates from 1900 (Fig. 4*B*) reveal that suicide rates vary similarly for males and females. An important exception occurred from the mid-1950s to 2000 where relative trends diverged between the sexes. Suicide rates for females rose rapidly (by 50% over about 15 y, and then declined by 50% between 1970 and 2000, only to rise once again. In contrast, rates were largely stable for males until the 1990s. Subsequently, variability in male and female suicide rates broadly resumed the pre-1950s similarity. In contrast to the findings above about the consistently declining rates for women after 38, the indexed “bump” suggests that the changing roles and norms that came at the beginnings of the Women’s Movement in the United States may have disrupted the social order for women expecting rapid change.

### Suicide Deaths by Hanging Have Risen Consistently over the Last 20 y.

**Fig. 5.**
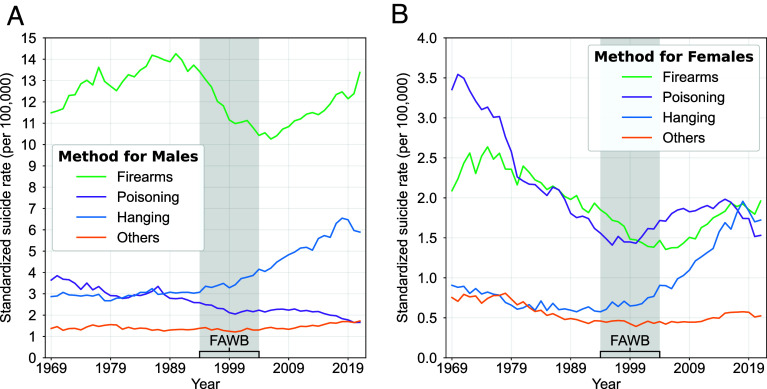
Age-standardized suicide rates by method for males and females, 1969–2021. Suicide rates are shown for each major method of death for *A* males and *B* females. The period of the Federal Assault Weapons Ban (FAWB) from 1994–2004 is annotated with shading. ICD codes used in encoding the causes of death can be found in *SI Appendix*, Table S2.

Traditionally, firearms, hanging, and poisoning represent the most common and lethal means of suicide, with firearms representing over half of deaths, especially for males (Fig. 5) (35–37). As Shields et al. noted, hanging was the most common cause in 1860, replaced by poisoning in 1900 (37). And by 1910, firearms represented the most common method. However, our MCOD data reveal a substantial, sustained rise in suicide by hanging beginning in the early 1990s, coinciding with the new Federal Assault Weapons Ban (~1994–2004). During this period, suicide deaths were at a low, with the most recent upward mortality trend beginning in 2003 (Fig. 1). This rise in hanging deaths, which became a more prominent method among females than males (though the rates remain lower among females in absolute terms), has been sustained to the present among both sexes.

### Suicide Rates Are Higher in Rural and Urban Areas But Persistently Lower in Large Metro Areas.

**Fig. 6.**
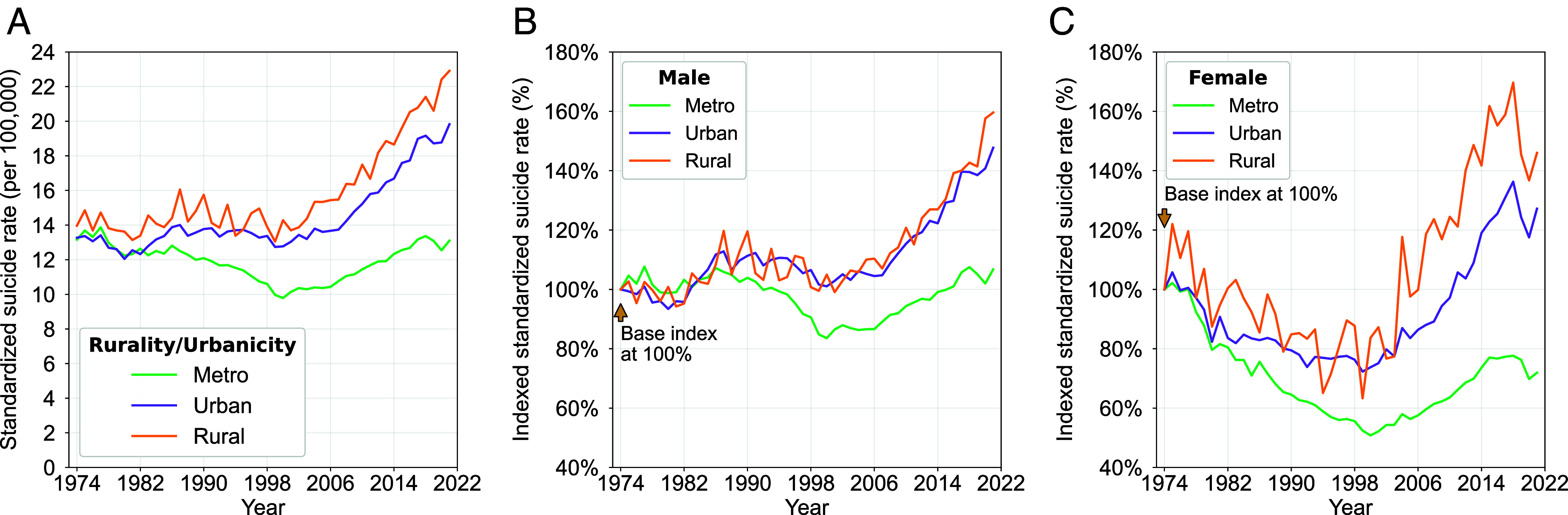
Age-standardized and indexed suicide rates by degree of urbanicity/rurality. Age-standardized rates are shown for metro, urban, and rural counties as defined by RUCC in *A*. Corresponding indexed rates, with 1974 as the baseline, are displayed for *B* males and *C* females.

Reports from the CDC and National Rural Health Association have recently highlighted higher suicide rates in rural areas, especially among youth (38, 39). Kegler et al. reported that between 1999 and 2015, higher suicide rates were found in “less urbanized” areas, with the gap in rates widening (40). Focusing on three geographical levels (i.e., metro, urban, and rural) over time, our analyses using MCOD data from 1974–2021 (Rural-Urban Continuum codes (RUCC) codes were developed in 1974) showed less variation in suicide rates across the three levels in 1974 and divergence over time (Fig. 6*A*).

Overall rates are highest in rural counties, intermediate in urban counties, and lowest in metro counties. Using indexed rates, urban and rural rates continued to covary for males (Fig. 6*B*) while they diverged for females, with rates for rural females showing the greatest increase (Fig. 6*C*). More consistently, rates for both males and females in metro areas have been lower since the early 1980s. This both supports and contradicts the well-known findings on “deaths of despair” (3) which emphasized the potential role of economic forces and profiles that are typically seen in rural areas including closing industries and lower levels of education. Regarding suicide, this is further supported by greater access to lethal methods (e.g., firearms, pesticides), lower access to mental health services and, most recently, political polarization. To the contrary, however, our results indicate that—especially for men who were of the greatest concern in deaths of despair—urban and rural suicide rates have covaried. And, if political polarization underlies a divergence from that trend, it is women, not men, in rural areas that have departed from that pattern (39, 41).

### A Note on Race and Suicide.

Efforts to study long-term race and ethnicity trends in suicide are important but empirically problematic. Data available from censuses, surveys, and vital records reflect prevailing legal, social, and administrative standards. Accordingly, racial classifications have been fluid with standards established in one era superseded by successively newer standards (42–44). While suicide trends have been shown to vary by racial groups in shorter time periods with similar data protocols, (45–47) assessing long-term trends is difficult if not impossible. This includes the important work of examining the age structure of suicide trends in race. We are unable to confidently portray suicide trends by race over such a long historical period. Most of what we reliably know about US race-based suicide trends covers only the last 25 y (45, 47, 48). However, even these studies question the “Black suicide paradox” (49). That is, given the economic and socially stigmatized status of Blacks in the United States, and, even given similar risks as Whites, the expectations that crude and/or age-standardized.

**Fig. 7.**
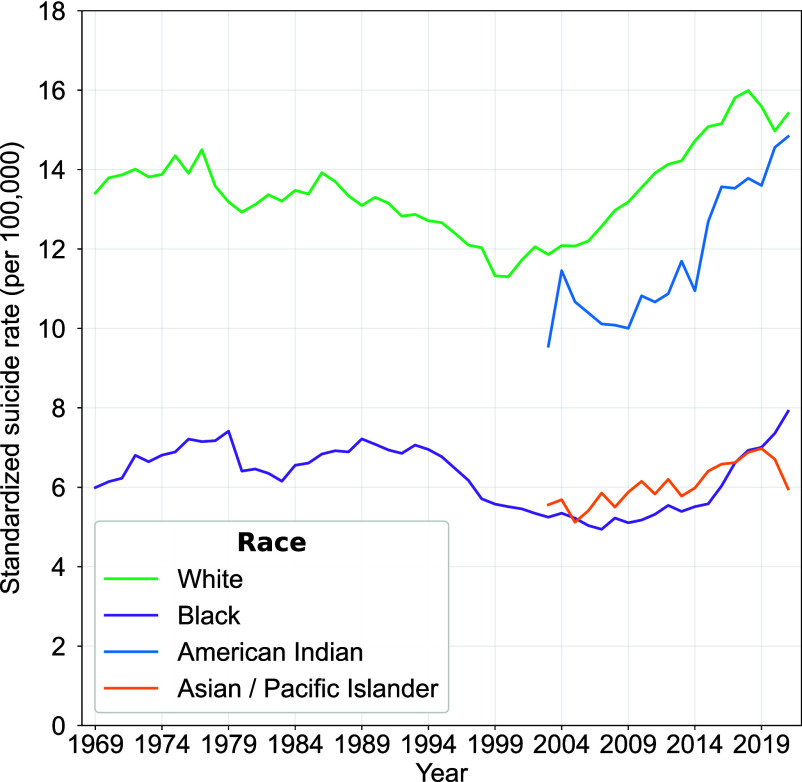
Age-standardized suicide rates by race, 1969–2021 The use of race categories in the underlying datasets is unstable over time. Data for four races only become available after 2003.

US suicide rates for Blacks should be higher does not bear up. Studies that attempt to take racial misclassifications into account continue to document the persistence of the paradox even as the racial suicide gap has decreased somewhat (50–52). Given this caution, Fig. 7 provides trends in age-standardized suicide rates between Whites and Blacks (MCOD, 1969–2021) and more detailed categories (MCOD: White, Black, American Indian, Asian, or Pacific Islander) since 2003.^†^ Overall, the suicide rates for Black and White individuals tend to trend together, with the rate for Whites being consistently higher. For the most recent years, rates for American Indian/Alaska Native persons are slightly lower than for Whites while the Asian/Pacific Islander rates are much lower, trending with suicide rates for Blacks, except in the most recent years.

## Discussion

Suicide has been both intensively and extensively investigated since ancient times. Yet, the roots of suicide remain indistinctly understood. From basic psychological approaches that focus on individuals’ psychological traits or stress, (53–57) to the more recent role of biological vulnerabilities (e.g., differences in brain structure or function; (58) genetic variants/risk scores), (59, 60) the targets are numerous. Our focus was different and specific—examining the consistency of the association of social, geographic, and temporal factors over time to ask whether stability or periodicity better characterizes the association of sociodemographic factors with suicide risk.

Our results indicate that the temporal variability in suicide rates is strongly associated with social, geographical, and/or cultural forces. Joinpoint regressions revealed a dynamic quality characterized by cyclical fluctuations within short timescales, an unusual pattern in human health. Without denying their role, if suicide were entirely determined by biological, psychological traits, or interpersonal crisis, then we would not expect to observe this cyclicality over the study period. Even traits under very strong selection pressures such as lactose intolerance or high-altitude adaptation to low oxygen likely took several thousand years to emerge (61, 62). Repeated doubling or halving of suicide rates over 10 to 25 y intervals suggests responsiveness to external forces that vary over similar timescales. This supports leading multifactorial theories that characterize suicide as the outcome of interactions among individual psycho-biological vulnerabilities, social conditions, and access to means (63). While generally accepted in prior research, temporal considerations are difficult to incorporate in many cross-sectional or truncated time-series research designs, and arguments for the primacy of social relative to biological factors continue (41).

In our data, we do not observe the kinds of sustained decreases that tend to characterize disease or injury classes where interventions have durably reduced rates over time. Our findings strongly suggest that the substantial and standard public health efforts undertaken to prevent suicide at the individual level may have failed for lack of understanding the powerful influences of larger contexts on suicide. Precision medicine has tended to see greater tailoring on more and more refined individual biological levels, augmented by in silico approaches (e.g., genetics, social determinants of health, computer simulation, respectively) (64). Our data foreground the need to consider larger contexts in which individuals experience suicide risk and reduce risk by introducing initiatives that are tailored to time and place (65). It may be of no small consequence that the US Air Force Suicide Prevention Program (AFSPP) was essentially a multilevel approach to engage and change a whole community. While the evaluators of the long-term effectiveness of this program pointed to extensive and effective monitoring, the AFSPP’s multilayered and overlapping approach was based on changing the view of suicide (and mental illness) from a medical problem to a service-wide community problem (66).

Further, a persistent, long-term minimum bound or baseline to US suicide rates (from approximately 9 to 11 deaths per 100,000 population) echoes Durkheim’s early claim of a society’s “natural” or “definite” aptitude for suicide only when the social structure is constant (1). While baseline prevalence in a society or social group may be determined by psycho-biological factors, changes in the social environment, broadly defined, can modify risk, producing temporal variation in suicide rates. Many known socioeconomic cycles follow multidecadal timeframes, such as generational, political, Kuznets, Kondratieff, and economic supercycles, some of which have been applied to suicide (30, 67–70). We contend that the dynamics underlying suicide deaths are more complex than any one cycle, though the inception of each major uptrend in US suicide rates (1920s, 1960s, and 2000s) may share common socioeconomic characteristics (e.g., significant, youth-driven shifts in social norms and values, economic boom-bust phenomena, or technological advancements and disruption). Future work to increase effectiveness of suicide prevention and treatment will benefit from understanding and incorporating the impact of larger social contexts in the face of individual-level risk factors. Models that decompose baseline, trend, and seasonality elements may identify specific contextual causes of cyclical trends. This would be no small challenge for researchers given the limits of data on individual suicides, even with National Violent Death Reporting System (NVDRS) innovations (9).

Over the last 50 y, suicide has been increasing among younger people, reported over time in a range of studies that focus on associations based on shorter time intervals than those in our study. For instance, the upswing since approximately 2010 gave rise to studies linking suicide to increased depression, screen time, and social media use, (71) while the upswing seen in the 2000s was associated with use of antidepressants, (72) and the earlier 1950s-1980s uptrend to increased drug and alcohol use (73). However, indexing long-term rates shows that each shorter-term increase was, in fact, part of a much longer, sustained trend, where the rate of change in suicide death among youth *and* young adults up to 35 y of age markedly diverged from older adults in the mid-1950s and has never since converged to that of the overall population. A sustained shift of this nature that straddles multiple age groups and cohorts suggests one or more structural factors affecting the experience of emerging and young adulthood. For instance, this pattern is most prominent among 5 to 14 y-olds, suggesting a possible alignment with the well-documented decrease in both boys’ and girls’ pubertal age, implicated in negative psychological outcomes (74). Even more to the point, a recent, influential theory suggests that two contextual forces in childrearing have negative mental health effects—the decline of the play-based childhood and rise of the phone-based childhood (75). This idea is bolstered by a long history in philosophy, sociology, and psychology that rising expectations both characterize social change, and the failure of realized gains to meet those expectations have always had dire consequences. Social changes affecting relational and behavioral functioning and their consequences have levied a particularly heavy burden on contemporary youth (1, 76, 77). Importantly, this dynamic has led to an overall *convergence* of crude rates among different age groups over the last 40 y (70). Prior to the 1980s, age strongly discriminated risk, but accelerating rates among persons under age 35 coupled with decelerating rates among those 35 and older appear to have caused this distinction to largely disappear. On the one hand, while absolute rates remain marginally higher in older cohorts, our findings challenge narratives based on shorter-term data such as the spotlight on “deaths of despair” among middle-aged non-Hispanic White US residents (3). On the other hand, our findings buttress fragmented, shorter time-series, and international data showing cohort effects that suggest “imprints” on the age-suicide connection that likely drive some of our observed rate dynamics (78–80). While age is “ahistoric,” cohort analyses spotlight historical context: Individual suicide risk depends not only on a person’s age but also on the historical period in which they lived. The emphasis on cohort differences highlighted here are also closely connected to the impact of historical or period effects that have occurred during the past century. Accordingly, future work on formal age-period-cohort (APC) analyses of suicide rates spanning many decades are warranted but their implementation is beyond the scope of this paper, especially given the methodological nuances and challenges of estimating APC models (81–83).

The common preconception that US male suicide rates are higher than for females appears to hold since 1900 in the United States. Yet, by indexing rates over a century, our findings reveal three hidden insights. First, the temporal volatility of sex-specific rates is similar *except* for the period from 1950–2000. That temporary spike in female suicide rates differs from conclusions in early research that male rates are more sensitive to context (84). Our observation is more congruent with recent research suggesting a critical nuance: Male rates may be more sensitive to income inequality while female rates are more sensitive to fluctuations in social capital, or perhaps in this case, to changing sociocultural roles and resources that may have arisen as the US Women’s Movement emerged (85). In line with the discussion about age shifts above, the idea that macrolevel social change produces uncertainty, rising expectations and turmoil, the indexed “bump” reported for female suicide in the early 1970s points to that same set of forces. This has been clearly documented in the case of mass suicides and in other historical epochs of rapid change such as industrialization. In sum, suicide has widely and often been seen as the “penalty” for normative disruption, even when that change represents progress (women’s movement), nevermind when it has no other beneficial consequences (increased pressure in childhood). Second, the end of this period coincides with a second remarkable phenomenon among females. From the late 1990s onward, our findings on suicide method reveal a strong and sustained trend away from the long-held female selection of poisoning to suffocation (e.g., hanging). This trend has also been documented globally, where it has received more attention (86–88). This shift to hanging among females has occasionally been reported, but its lethality has not been a prominent target for intervention (89). Third, our results suggest that the recent increase outside of large, metro areas has been for rural women, not rural men nor urban residents as a whole. While the rates of increase of these smaller units and metro areas have moved in tandem, the lack of a focus on women in rural areas stands as a critical gap in attention and in understanding what is happening in rural areas generally. If, as some have suggested, political polarization, isolation, and despair are the roots of rural suicide, then the focus on women must be at the center of further research.

Some recent work documented higher and rising rates in rural areas for both the United States and cross-nationally (90–92). While we cannot examine these effects in the United States prior to the 1970s given data limitations, our analyses reveal three striking findings. First, since the late 1990s, US suicide rates have been increasingly higher in rural *and* smaller urban areas than in larger metro areas, suggesting that future research should examine suicide risks across the metropolitan–rural gradient and abandon the binary urban/rural typology used in many prior studies. Second, the post-1998 growth of that suicide risk gap—between metro areas and both rural and urban areas—has been more dramatic for females than males. Third, as suicide risks have generally risen nationally since the late 1990s, living in a US metro area has, in fact, emerged as a protective factor against suicide, especially for females. This implies that future research and prevention efforts should be extended to encompass not only rural areas but also smaller cities and exurbs, focused both on males and females. Others have documented widening urban–rural disparities in US suicide rates from 1970–1997 and 2001–2015 (48, 93). If data improvements become available, it will be important to consider the role of migration and how movement between locations can help to understand spatial population sorting. For example, suicides occurring in metro areas may arise from migrants who grew up in rural communities. Finally, our analyses have targeted geographic patterns based upon a county-level trichotomy of metro–urban–rural counties. The newly available mortality data released by the CDC containing indicators of location information at greater geographic resolution provide opportunities for revealing new sociospatial patterns of suicide risk.

We acknowledge several data limitations that may affect our findings. In 2002, the Institute of Medicine (now National Academy of Medicine) concluded that suicide statistics are “fraught with inaccuracies” (17, 94). Foremost among them are those that stem from the cause of death classification process. Some have raised concerns that the male–female disparity in rates may be attributable to the use of less violent means among females (52). Others reflect concerns for potential and often unacknowledged biases and influences from religious or political officials as well as stigma to the family (17, 95). Overall, Tøllefsen et. al.’s review noted a lack of systematic and broad-based assessment of the reliability of suicide statistics (21). Many efforts that support claims of bias involved case studies of types (e.g., railroad suicides) (96) or cross-national comparisons (97). Given the US’s patchwork of state-based medicolegal systems, concerns center around disagreement on the burden of proof among medicolegal officials and the consistency of reporting medical examiners. For example, critiques targeted nonmedical death investigators (e.g., funeral directors in Nevada) who lacked the expertise and skills to discriminate accident from suicide. Among those few studies that organized criticisms and deployed either in-depth ethnography or measurement models with a number of techniques to control for misreporting, systematic bias was in evidence (e.g., potential undercount of young male equivocal car crashes as accidents rather than suicide). However, both qualitative and quantitative analyses concluded that the variation in and absolute levels of suicide was greater than such errors, allowing for reasonable conclusions from the analysis of suicide rates (18, 95). In addition, past analytic findings were contrary to expectations—scientifically trained medicolegal official were *less* likely to code a suspicious death a suicide than others, requiring a greater burden of proof to classify a suspicious death as a suicide (19, 98). On changing ICD coding, analyses examining revisions from ICD-6 to ICD-9 did not appreciably shift rates of suicide in the United States or in other nations (22). More recently, the transition from ICD-9 to ICD-10 did not drastically alter coding for US suicide rates (23). We are unaware of any systematic assessment of suicide coding discontinuities per se across ICD-1 through ICD-7 revisions although some have shown that they exist across very broad diseases classifications (99). Specifically, using nested classifications of causes of death (where some ICD codes could be “nested” within other ICD revisions due to code changes), suicide was fairly consistently reported across revisions [see Table 2 in ref. 99]. Our own work has indicated that “misrepresenting [suicide] has little effect on the relationship between suicide rates and indicators of concepts in sociological theories of suicide” (19). More curious is the difference in the greater temporal volatility of suicide in the early years of our sample (see Fig. 1, 1900–1940). Of course, the number of states that made up the United States changed over that period as did our population, affecting both the size of our “sample” and, potentially, some unmeasured factor that translated into greater shifts. For the early decades, we rely on data generated as it was established at the start of the 20th century. As such, these statistics represent the official rates for participating states. Since vital record keeping was still evolving, there may be biases in the data collection for these early registration years. If there were initial categorical inefficiencies, one might expect the rates to be rising, as they did initially, but continue throughout, which they did not. Neither necessarily align with convulsive social epochs such as the Spanish Flu pandemic or a decrease as newer states, less experienced with vital registration joined the union. For our own purposes, we presented the suicide rate for the original 12 states until 1960 when the continental US states were fully formed. The close correspondence between the suicide rates across the entire timeline suggests that whatever cause lies at the root of the early volatility is not due to the smaller sample or unique suicide profile of the original states or the sequential addition of new states.

## Conclusion

According to Marques et al., precision medicine has revolutionized healthcare “by individualizing diagnostics and treatments according to each patient’s uniquely evolving health status” which includes individual genetic, environmental, and lifestyle differences. They state that “the goal of precision medicine is to target the “five rights”: the right patient, the right drug, the right time, the right dose, and the right route” (64). Yet, even this conceptualization of “environment” leans toward the typical idea of sociodemographic characteristics and the social determinants of health, an innovative but limited step. They contrast this with the older concept of personalized medicine which included genetic makeup, beliefs, preferences, knowledge, and social context. Precision medicine also misses the fundamental point of the most successful suicide reduction program to date. The US Air Force Suicide Prevention Program which, during its 12 y of full application, was built on a shift from the individual to community, connectedness, and consistent implementation. The present work in long-term suicide dynamics shows that suicide death in the United States is an unusually cyclical phenomenon that has been responsible for ~400,000 excess deaths over the last half century. Within this overall pattern, higher and durable relative risk increases for younger people, females, and those in rural and smaller urban areas have been occurring over the last 50 to 70 y. Since suicide theories based on individual psychological or biological traits do not account for these multidecade risk cycles and shifts, an inescapable hypothesis is that structural changes in the social and economic context likely number among the major driving forces.

### Implications.

Our work strongly suggests that social and economic contextual factors influence pronounced cyclicality in suicide rates, presenting important implications for further research and intervention development, policy planning, and resource allocation. Suicide is always on a 10 to 25 y upward or downward trend. At a minimum, this background periodicity should be accounted for when testing the effectiveness of interventions to avoid misattributing success or failure. In essence, results should be detrended. However, our observations also single out areas where new investigation is needed. Despite the early and intensive focus, we still lack robust predictive models of suicide risk that are prerequisites for developing risk stratification tools to target existing preventive interventions (100). Influential studies have called for an enlargement of risk factors under consideration beyond individual characteristics (6). Uncovering the social bases of cyclicality in suicide rates, rising hanging deaths, the protective effect of living in metro areas and the contextual determinants of the increasing socioeconomic vulnerability for youth and females since the 1950s as well as age and especially cohort risk differences are all required to inform and strengthen individual-level models and theories of suicide death (65, 101). Ultimately, determining the contextual factors driving the long term trends that we identify in the present work will also suggest the synthesis and timing of socioeconomic interventions at local, community, and national levels (66, 102).

## Supplementary Material

Appendix 01 (PDF)

## Data Availability

The analysis represented in this study received approval to use restricted microlevel mortality data from 1969–2021 from the Centers for Disease Control and Prevention (CDC) called the Detailed Multiple Cause of Death (MCOD) Research Files. All investigators may submit applications to the CDC to request access to these restricted data by completing the Project Review Form (https://www.cdc.gov/nchs/data/nvss/nchs-research-review-application.pdf) (13) and emailing it, along with supporting documents, to nvssrestricteddata@cdc.gov. More details can be found at https://www.cdc.gov/nchs/nvss/nvss-restricted-data.htm (12). Code used to parse the raw MCOD data files and replicate our presented analyses were implemented in Python and can be found at https://github.com/delacylab/Century_of_Suicide/ (103).

## Footnotes

^*^Age standardization over a very long time period such as we analyze here tends to distort the time series since a single timepoint is customarily used to perform the standardization.

^†^In MCOD, race was classified as White, Black, and Others in 1969–2002. In 2003–2020, the classification became White, Black, American Indian, and Asian or Pacific Islander. Starting 2021, the classification allowed for mixture of races, resulting in 40 possible classes. Bridging was performed from 40 races to 4 races to allow for a better comparison in Fig. 7.
