# Supplementary material for: A century of suicide: Insights from long-term data in the United States
Source: Proc Natl Acad Sci U S A. 2026 Apr 27;123(18):e2519951123. doi: 10.1073/pnas.2519951123 (PMC13142972; doi:10.1073/pnas.2519951123)
Supplement: Supplementary file 1 — Appendix 01 (PDF) [file pnas.2519951123.sapp.pdf]

Supporting Information for
A century of suicide: insights from long-term data in the United States

Nina de Lacy[1,2], Wai-yin Lam[1], Timothy Collins[3], David Danks[4], Fernando A. Wilson[5], Ken Smith[6,7], Bernice A. Pescosolido[8,9]

1 Department of Psychiatry, University of Utah, Salt Lake City, UT 84112
2 Huntsman Mental Health Institute, Salt Lake City, UT 84108
3 School of Environment, Society, & Sustainability, University of Utah, Salt Lake City, UT 84112
4 Halicioğlu Data Science Institute, Department of Philosophy, School of Global Policy & Strategy, University of California San Diego, La Jolla, CA 92093
5 Matheson Center for Health Care Studies, University of Utah, Salt Lake City, UT 84108
6 Department of Family and Consumer Studies, University of Utah, Salt Lake City, UT 84112
7 Huntsman Cancer Institute, Salt Lake City, UT 84112
8 Department of Sociology, Indiana University, Bloomington, IN 47405
9 Irsay Institute, Indiana University, Bloomington, IN 47405

Corresponding Author:
Bernice A. Pescosolido, Sociology & Irsay Institute, Indiana University
Morrison Hall 313, 1165 E. Third Street, Bloomington, IN 47405
Email: pescosol@iu.edu | Phone: (812) 855-6213 | Phone: (812) 855-6256 assistant

This PDF file includes:

Figure S1
Tables S1 to S3

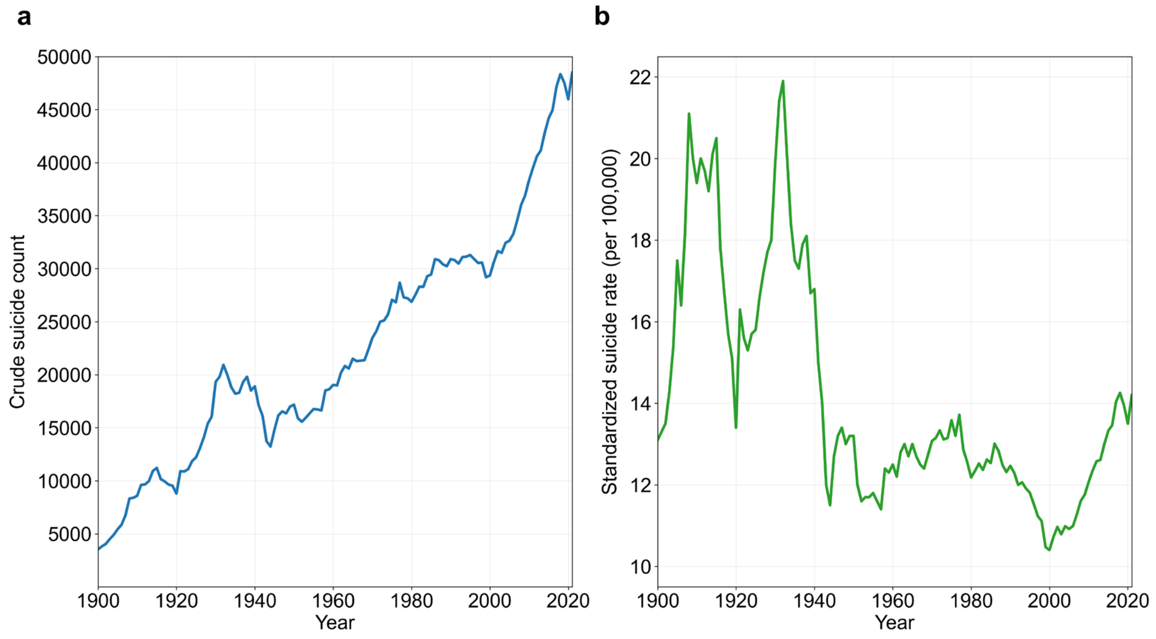

Fig. S1. Crude suicide counts and age-standardized suicide rates, 1900-2021

Table S1. Total annual suicide rates recorded in the Multiple Cause of Death files provided by the National Center for Health Statistics, 1969-2021

1969: 22,364	1970: 23,480	1971: 24,092	1972: 25,004
1973: 25,118	1974: 25,683	1975: 27,063	1976: 26,832
1977: 28,681	1978: 27,294	1979: 27,206	1980: 26,869
1981: 27,547	1982: 28,312	1983: 28,295	1984: 29,286
1985: 29,453	1986: 30,904	1987: 30,796	1988: 30,407
1989: 30,232	1990: 30,906	1991: 30,810	1992: 30,484
1993: 31,102	1994: 31,142	1995: 31,284	1996: 30,903
1997: 30,535	1998: 30,575	1999: 29,199	2000: 29,350
2001: 30,622	2002: 31,655	2003: 31,484	2004: 32,439
2005: 32,637	2006: 33,300	2007: 34,598	2008: 36,035
2009: 36,909	2010: 38,364	2011: 39,518	2012: 40,600
2013: 41,149	2014: 42,826	2015: 44,193	2016: 44,965
2017: 47,173	2018: 48,344	2019: 47,511	2020: 45,979
2021: 48,483			

Table S2. International Classification of Disease (ICD) codes used to ascertain causes of death

Underlying cause of death	ICD-8 (1969-1978)	ICD-9 (1979-1999)	ICD-10 (2000-2021)
(a) Suicide by firearms or explosives	E955	E955.0-9	X72-X75
(b) Suicide by poisoning	E950.0-9, E951, E952.0-1, E952.9	E950.0-9, E951.0-1, E951.8, E952.0-1, E952.8-9	X60-X69
(c) Suicide by hanging, strangulation, or suffocation	E953	E952.0-1, E952.8-9	X70
(d) Suicide by other methods	All codes in E950-E959 not included in (a), (b), or (c).	All codes in E950-E959 not included in (a), (b), or (c).	All codes in U03, X60-X84, and Y-87 not included in (a), (b), or (c).
(e) Heart attack	410	410	I21 and I22
(f) Homicide	E960-E970	E960-E970	X85-X99, Y01-Y09, and Y871, U01, and U02
(g) Motor vehicle traffic crashes	E810-E819	E810-E819	V02.1, V02.9, V09.2, V12-14 (.2-5), V13.2-V13.5, V19.4-V19.6, V20-28 (.3-5 and .9), V29-79 (.4-9), V80.3-V80.5, V81.1, V82.1, V83-V86 (.0-3), V87.0-V87.8, and V89.2
(h) Overdose	E850-E859, E950, E962, and E980	E850-E858, E950, E962, and E980	X50-X44, X60-X64, X85, and Y10-Y14 with at least one recorded multiple causes of death from T36-T50.

Table S3. Annualized change and percentage change of segments of the joinpoint regression trendline in Figure 1, 1900-2021

Period	Absolute annualized change (deaths per 100,000 per year)	Percentage change per year
1900-1908	+0.63	+4.60%
1908-1915	-0.12	-0.73%
1915-1920	-1.12	-8.37%
1920-1932	+0.52	+4.03%
1932-1944	-0.45	-3.29%
1944-1960	-0.06	-0.60%
1960-1977	+0.15	+1.33%
1977-1980	-0.25	-2.01%
1980-1986	+0.16	+1.28%
1986-2003	-0.13	-1.14%
2003-2018	+0.27	+2.21%
2018-2021	-0.11	-0.74%
